# Bilateral Parsonage-Turner Syndrome After Initial Unilateral Presentation: A Case Report

**DOI:** 10.7759/cureus.6422

**Published:** 2019-12-19

**Authors:** Brendan Lindgren, Dustin Rivers, Jeffrey Clark

**Affiliations:** 1 Neurology, Northwell Health, Riverhead, USA; 2 Neurology, Baylor Scott and White, Temple, USA

**Keywords:** brachial neuritis, shoulder pain, neurogenic amyotrophy, parsonage-turner syndrome, idiopathic brachial plexopathy, acute brachial neuropathy, brachial plexitis, functional disorders, upper extremity weakness, shingles vaccine

## Abstract

Parsonage-Turner syndrome (PTS) is a clinical syndrome characterized by rapid onset of upper extremity pain typically followed by varying degrees of weakness and atrophy. In this case, we discuss a 54-year-old female who developed severe right upper extremity pain soon after receiving a shingles vaccine, which was then followed by weakness and atrophy. Thorough medical workup was unrevealing, as it was too early to see electromyography (EMG) changes. Nine months later she presented to the hospital again with a similar presentation of the contralateral upper extremity. EMG findings at that time were supportive of a PTS diagnosis. This report discusses the clinical variability, etiology, and treatment of PTS, as well as some diagnostic complexities and how they were overcome to diagnose this patient.

## Introduction

Parsonage-Turner syndrome (PTS), brachial neuritis, idiopathic brachial plexopathy, and neurogenic amyotrophy are some of the many terms that refer to the same clinical syndrome that is characterized by rapid onset of upper extremity pain typically followed by varying degrees of weakness and atrophy. Beyond that, the clinical syndrome is highly variable. The distribution of nerves affected can vary from anything between an isolated mononeuropathy in the upper extremity and the entire bilateral brachial plexus, possibly in combination with the lumbosacral plexus, phrenic nerve, cranial nerves, and/or other peripheral nerves [1]. With that in mind, nearly any distribution of weakness or sensory symptoms can be found, though some distributions are certainly more common than others. While an initial period of pain is almost invariably present and lasts around four weeks on average, it can last less than one week in 5% or greater than two months in 10% [2]. Recovery of strength also varies considerably from patient to patient. By the end of year 3, most patients recover 80%-90% of their strength, but greater than 70% are left with residual weakness and exercise intolerance [1]. The exact cause of this disorder is unknown, but greater than 50% report an immunologic event before the episode, certain mutations (SEPT9) appear to make patients more prone to it, and nearly 10% are preceded by unusual exercise [1].

## Case presentation

A 54-year-old right-hand dominant female chef at a local university presented with severe bilateral upper extremity weakness and sensory changes. Approximately nine months earlier, she had developed right upper extremity (RUE) pain a few days after receiving a shingles vaccine, which was quickly followed by RUE weakness. She saw an outside neurologist at that time, and after an extensive workup that included magnetic resonance imaging (MRI), electromyography (EMG), nerve conduction studies (NCS), and lumbar puncture (LP), no organic cause was discovered, and based on her clinical examination a functional disorder was suspected (giveway/collapsing weakness). Five months later, a similar series of symptoms occurred in the left upper extremity (pain followed by weakness). No further workup was done at that time, and she did not receive treatment due to the belief that her symptoms were psychogenic. Despite spontaneous resolution of pain, she presented to the emergency room for persistent weakness four months later (nine months after the initial event involving the RUE).

On physical examination during her most recent admission, profound symmetric weakness was noted in her bilateral upper extremities. The weakness was more severe proximally (2/5 strength in deltoids; 3/5 biceps, triceps, wrist extensors, wrist flexors, and intrinsic hand muscles; 4/5 finger flexors and extensors), and significant atrophy of the bilateral shoulders was noted. Her upper extremity reflexes (biceps, triceps, brachioradialis) were absent bilaterally, with preservation of her lower extremity reflexes. Sensation to pinprick, light touch, vibration, and temperature were diminished symmetrically up to the shoulder in her bilateral upper extremities.

During her most recent admission, she received an evaluation that included MRI with gadolinium of the brain, cervical spine, thoracic spine, and brachial plexus, along with LP (Table 5), EMG (Tables 1, 2), and NCS (Tables 3, 4). The MRI brain, cervical spine, thoracic spine, and LP revealed no contributory findings to her clinical symptoms. EMG of her bilateral upper extremities revealed evidence of an active denervating process involving C5 through T1 distribution in both upper extremities. No genetic testing was done, but she denied any family history of similar symptoms. Results in Tables 1-5 are all from her final admission.

**Table 1:** Electromyography F Wave Amp = amplitude, Lat = latency

Nerve	Min M Lat (ms)	Max M Lat (ms)	Mean M Lat (ms)	Min M Amp (mV)	Max M Amp (mV)	Mean M Amp (mV)	Min F Lat (ms)	Max F Lat (ms)	Mean F Lat (ms)
R Ulnar	2.81	2.97	2.89	7.31	7.71	7.45	27.86	28.23	28.00
R Tibial (foot)	5.26	5.36	5.31	7.72	8.12	7.92	50.26	52.50	51.41

**Table 2:** Needle Electromyography Amp = amplitude, Dur = duration, Fasc  = fasciculations, Fib = fibrillation, H.F. = high frequency, IA = Ia fibers, LD/LDP = long duration/long duration potentials, MUAP = motor unit action potential, N = normal, PPP = polyphasic potential, PSW = polyspike wave, RIFP = reduced interference pattern; EMG, electromyography

EMG Summary Table												
			Spontaneous					MUAP			Recruitment	
Muscle	Nerve	Roots	IA	Fib	PSW	Fasc	H.F.	Amp	Dur	PPP	Pattern	Comments
R. Deltoid	Axillary	C5-C6	N	2+	2+	None	None	N	N	N	Markedly RIFP	LDP
R. Biceps brachii	Musculocutaneous	C5-C6	N	2+	3+	None	None	N	N	N	N	No Vol units
R. Triceps brachii	Radial	C6-C8	N	1+	2+	None	None	N	N	N	N	None
R. Extensor carpi radialis longus	Radial	C5-C6	N	2+	2+	None	None	N	N	N	Markedly RIFP	LD
R. First dorsal interosseous	Ulnar	C8-T1	N	2+	3+	None	None	N	N	N	RIFP	LD/LDP
R. Quadriceps	Femoral	L2-L4	N	None	None	None	None	N	N	N	N	None
R. Tibialis anterior	Deep peroneal (fibular)	L4-L5	N	None	None	None	None	N	N	N	N	None
R. Gastrocnemius (medial head)	Tibial	S1-S2	N	None	None	None	None	N	N	N	N	RIFP
L. Deltoid	Axillary	C5-C6	N	1+	2+	None	None	N	N	N	Single unit interference pattern	None
L. Biceps brachii	Musculocutaneous	C5-C6	N	3+	3+	None	None	N	N	N	Single unit interference pattern	None
L Triceps brachii	Radial	C6-C8	N	1+	2+	None	None	N	N	N	Markedly RIFP	LDP
L First dorsal interosseous	Ulnar	C8-T1	N	2+	2+	None	None	N	N	N	RIFP	NL LD/LDP
R. Cervical paraspinals	Spinal	C4-C8	N	None	None	None	None	N	N	N	N	Poor relaxation
L. Cervical paraspinals	Spinal	C4-C8	N	None	None	None	None	N	N	N	N	Poor relaxation

**Table 3:** Sensory Nerve Conduction Studies

Nerve/Sites	Peak (ms)	Duration (ms)	Amplitude (µV)	Distance (cm)	Velocity Peak (m/s)
R Median – Digit II					
Wrist	4.74	3.96	14.5		
R Ulnar – Digit V					
Wrist	3.07	2.19	15.7		
B. Elbow	6.77	3.33	18.2	20	54.1
A. Elbow	8.02	4.58	17.2	7	56.0
R. Median – Ulnar – Palmar					
Median	2.60	1.41	65.3		
Ulnar	1.98	1.20	62.5		
R Sural – Lateral Malleolus					
Calf	2.92	2.24	21.5		

**Table 4:** Motor Nerve Conduction Studies NCV = nerve conduction velocity

Nerve/Sites	Onset (ms)	Peak (ms)	Duration (ms)	Amplitude (mV)	Area (mVms)	Notch	Delta (ms)	Distance (cm)	NCV (m/s)
R Median – Abductor Pollicis Brevis									
Wrist	6.15	9.01	14.06	3.5	14.3	Off	6.15		
Elbow	10.52	13.49	12.86	3.3	12.0	Off	4.38	22	50.3
R Ulnar – Abductor Digiti Minimi									
Wrist	2.97	6.41	15.16	6.9	29.7	Off	2.97		
B. Elbow	6.98	10.47	13.44	5.7	26.0	Off	4.01	21	52.4
A. Elbow	8.23	11.30	13.49	5.5	25.9	Off	1.25	7	56.0
R Peroneal – Extensor Digitorum Brevis									
Ankle	3.49	6.77	17.24	6.5	38.9	Off	3.49		
Fibular head	10.36	15.00	14.11	6.0	35.9	Off	6.88	35	50.9
Knee	11.82	16.72	16.77	5.9	36.0	Off	1.46	8	54.9
R Tibial (foot)									
Ankle	4.90	10.83	18.13	4.1	28.2	Off	4.90		
Knee	12.08	16.93	16.41	4.7	40.6	Off	7.19	40	55.7

**Table 5:** Lumbar Puncture Results CSF = cerebrospinal fluid, Ig = immunoglobulin, PCR = polymerase chain reaction, RBC = red blood cell, WBC = white blood cell

	Reference Range and Units	Result
Volume, CSF	mL	1.5
Clarity, CSF		Clear
Color, CSF		Colorless
RBC, CSF	/mcL	1
WBC, CSF	0-5/mcL	4
Lymphocytes, CSF	40%-80%	98
Monocytes, CSF	15%-45%	2
Differential based on, CSF	Cells	100
Glucose, CSF	50-80 mg/dL	66
Protein, CSF	15.0-60.0 mg/dL	23.0
Meningitis/encephalitis panel by PCR (CSF)	Negative	Negative
West Nile virus AB IgM, CSF	<=0.89 IV	0.02
IgG Monos, GM1	Negative	Negative
IgM Monos, GM1	Negative	Negative
IgG Asialo, GM1	Negative	Negative
IgM Asialo, GM1	Negative	Negative
IgG Disialo, GD1B	Negative	Negative
IgM Disialo, GD1B	Negative	Negative

## Discussion

PTS is a relatively rare diagnosis, with some estimates being as low as 1.64 per 100,000 population [3]. However, given the frequency of under-recognition and misdiagnosis, some have estimated that the true incidence is closer to 20-30 per 100,000 [2]. It is our belief that PTS was triggered in our patient secondary to the vaccine she received, though it is rarely possible to pinpoint a definite etiology. Many associations with PTS have been documented including infection, surgery, anesthesia, rheumatic disease, trauma, stressful exercise, pregnancy, radiation therapy, and vaccination [4]. Based on a thorough history, recent vaccination was the only association present in our patient. Given the lack of family history, our suspicion of hereditary PTS was low, though we cannot rule it out as a contributing factor as no genetic testing was done.

One of the difficult issues with diagnosing PTS is the lack of a reliable confirmatory test. The diagnosis is largely clinical, although there are a few tests, such as EMG, that can support the diagnosis. In the case of this particular patient, her first EMG was done too early to be of diagnostic value for her, as significant abnormalities often do not appear until three weeks after onset in PTS [5]. In most cases, the differential is broad and the history (especially time of onset) may not always be clear, so delaying EMG testing is not always reasonable. As suggested by this case, threshold for a repeat EMG in patients with persistent, recurrent, or worsening symptoms without a diagnosis should be low.

Evidence of effective treatment is limited, and significant spontaneous resolution is eventually seen in most patients, but one study suggests that oral prednisone given within the first month may shorten the painful interval and accelerate recovery in some patients [6]. In our patient, 38 weeks passed before the diagnosis was established, which was well beyond the suggested therapeutic window for prednisone mentioned above. This is actually shorter than the mean time of 43.8 weeks for diagnosis according to one review, although the median was significantly shorter at 10.5 weeks [2].

We treated this patient with IVIG and prednisone. There is no strong evidence as to how a patient with PTS should be treated in the chronic phase, but there is reported efficacy in case reports from the use of IVIG both as monotherapy and in combination with prednisone [7-12].

In the acute phase, the pain is often so intense that it is necessary to treat with opiates, tricyclics, or anti-epileptic drugs [13]. In the case of this patient, the pain had already resolved spontaneously, so there was no indication for pain management.

We also referred this patient to physical therapy, which is utilized for both range of motion and strengthening. Caution must be used in early reinnervation stages, as overloading injured muscles can retard axonal regeneration and muscle reinnervation [4]. Limited EMG follow-up testing may be utilized to demonstrate the extent of reinnervation and guide advancement of strengthening regimen, but is not absolutely necessary [4].

Although the diagnosis of PTS was significantly delayed in this patient, this does not necessarily mean that the suspicion of a functional disorder was incorrect. Functional disorders may be diagnosed based on positive findings, such as Hoover’s sign, drift without pronation, giveway weakness, and so on, and are not always a diagnosis of exclusion. With this in mind, it is important to consider that a patient may display these positive findings as part of a functional overlay to an organic neurologic disorder [14].

## Conclusions

In a patient presenting with shoulder pain followed by weakness, PTS should always be included in the differential diagnosis, especially if the initial workup does not reveal an obvious etiology. PTS is primarily a clinical diagnosis, and EMG findings are often unrevealing in the acute phase (up to three weeks). A functional disorder, even when correctly diagnosed secondary to positive findings as mentioned in the discussion, does not exclude an underlying organic neurologic disorder. In some cases, an organic neurologic disorder may even be considered a risk factor for a functional disorder. Current treatment options for PTS are limited, but there is evidence that oral prednisone within the first month may be beneficial, so a timely diagnosis is of increased importance. Given that EMG findings may not be apparent until three weeks after symptom onset, the threshold for ordering a repeat EMG should be low in patients without a strong diagnosis.
